# Divergences and gaps in life expectancy and health-adjusted life expectancy in Mexico: Contribution analysis of the Global Burden of Disease Study 2019

**DOI:** 10.1371/journal.pone.0293881

**Published:** 2023-11-06

**Authors:** Marcela Agudelo-Botero, Claudio A. Dávila-Cervantes, Omar Velasco-Calderón, Liliana Giraldo-Rodríguez

**Affiliations:** 1 Centro de Investigación en Políticas, Población y Salud, Universidad Nacional Autónoma de México, Mexico City, Mexico; 2 Facultad Latinoamericana de Ciencias Sociales (FLACSO), Sede México, Mexico City, Mexico; 3 Plan de Estudios Combinados en Medicina, Facultad de Medicina, Universidad Nacional Autónoma de México, Mexico City, Mexico; 4 Instituto Nacional de Geriatría, Mexico City, Mexico; University of Louisville School of Public Health and Information Sciences, UNITED STATES

## Abstract

**Introduction:**

Life expectancy (LE) and Health-adjusted life expectancy (HALE) are summary indicators that reflect a population’s general life conditions and measure inequities in health outcomes. The objective of this study was to identify the differences in LE and HALE by sex, age group, and state in Mexico from 1990 to 2019. Also, to evaluate whether the changes in HALE are related to sociodemographic indicators and indicators of access to and quality of health services.

**Methods:**

A secondary analysis was performed based on the Global Burden of Disease, Injuries, and Risk Factors Study (GBD). Data were obtained for LE (by sex and state) and HALE (by sex, age group, and state) for the years 1990, 2010, and 2019. The correlations between HALE with the Socio-Demographic Index (SDI) and with the Healthcare Access and Quality (HAQ) Index were estimated for 1990 and 2019 (by total population and sex).

**Results:**

LE and HALE had an absolute increase of 6.7% and 6.4% from 1990 to 2019, mainly among women, although they spent more years in poor health (11.8 years) than men. The patterns of LE and HALE were heterogeneous and divergent by state. In 2019, the difference in HALE (for both sex) between the states with the highest (Hidalgo) and the lowest (Chiapas) value was 4.6 years.

**Conclusions:**

Progress in LE and HALE has slowed in recent years; HALE has even had setbacks in some states. Gaps between men and women, as well as between states, are persistent. Public and population policymaking should seek to lengthen LE and focus on ensuring that such years are spent in good health and with good quality of life.

## 1. Introduction

Life Expectancy (LE) and Health-adjusted life expectancy (HALE) are indicators that reflect the population’s health conditions; they are closely related to the socioeconomic level and both the quality of and access to health services that people have [1–7]. These indicators are often used to identify gaps and inequities within and between countries. In order to calculate them, information is collected through censuses, vital statistics, or surveys, such as mortality, morbidity, and disability data, among others [1–7].

LE refers to the average number of years that a person at birth or at a certain age is expected to live, assuming that mortality rates remain constant at a given place and time [1–4]. This indicator represents the number of years that a person of a given age is expected to live [1–4]. Despite its widespread use, it is argued that LE does not consider the number of years a person can live with morbidity or disability. HALE, on the other hand, represents the number of years that a person of a given age is expected to live in good health, *i*.*e*., free of diseases or limitations [4–7]. Through this indicator, it is possible to add the quality aspect to the number of years lived [5, 7]. Accordingly, the years of life spent with “poor health” are the time spent without “good health” out of the total LE [1].

Knowledge on the differences in LE and HALE within the administrative/geographical divisions of a country, as well as the possible associated factors, are still scarce [8–12]. This is in spite of the fact that it is a useful resource for monitoring the burden of disease globally and for following up on international commitments such as those set out in the Sustainable Development Goals (SDGs). Although information on the LE trend at the national, state, and municipal levels is available in Mexico [13], only a few studies have focused on the geographical analysis of HALE [14].

Regarding LE, the National Population Council (in Spanish, Consejo Nacional de Población [CONAPO]) has official data at the national (since 1950) and state (since 1970) levels, as well as projections to 2050 [15]. According to this source, the LE at birth in 1950 was 47.34 years and is expected to be 79.62 years for both sex at the national level by 2050 [15]. The LE of Mexicans increased significantly until the beginning of 2000 [15, 16]. Since then, gains in life years have been stagnating, mainly due to the effect of homicides among young people and the evident mortality caused by chronic diseases such as obesity, diabetes mellitus type 2 (T2D), and chronic kidney disease [14, 17]. Between 2000 and 2020, the total LE at birth increased by only 0.40 years [15]. This increase was only 0.7 years for women and 0.33 years for men (calculated by the authors based on data from CONAPO) [15]. Furthermore, Gómez-Dantés et al. (2016) evaluated the HALE trend for Mexico and found that the difference between LE and this indicator was 9.7 years in 2013; Quintana Roo was the state where this gap decreased the most (from 10.4 years in 1990 to 10.2 in 2013), while it increased the most in Oaxaca (from 8.6 years in 1990 to 9.9 years in 2013) [14].

Data on LE and HALE are available internationally in the Global Burden of Disease, Injuries, and Risk Factors Study (GBD) [18–22]. An advantage of the GBD is that it uses standardized methods to estimate the different indicators, allowing geographical and temporal comparison. This helps to quantify more precisely the gains ―or losses― in health and the gaps between different population groups [4, 22, 23]. The latter is particularly relevant since the differences in these indicators reveal health inequities, which are unfair and affect the development of people’s capabilities [8, 24, 25]. The objective of this study was to identify the differences in LE and HALE by sex, age group, and state in Mexico from 1990 to 2019. Also, to evaluate whether the changes in HALE are related to sociodemographic indicators and indicators of access to and quality of health services.

## 2. Methods

### 2.1 Source of information and indicators

A secondary analysis was conducted based on the GBD 2019, which was developed by the Institute for Health Metrics and Evaluation (IHME) of the University of Washington. The study aimed to quantify the magnitude of health losses caused by diseases, injuries, and risk factors at the global, regional, national, and local levels [22, 23]. The GBD provides information on various health indicators, including prevalence, incidence, mortality, years of life lost (YLL), years lived with disability (YLD), and disability-adjusted life years (DALY) [22, 23]. In 2019, the study covered 204 countries and territories and 369 diseases and risk factors [22, 23].

LE at birth was obtained from mortality data at specific ages to determine the survival probability at a certain age [19–23]. This was done by means of life tables that were used to create hypothetical cohorts [19–23]. The data used to produce these tables came from civil registration systems, household surveys, censuses, and disease and demographic surveillance systems, among others [22]. From this information, two indicators were calculated that reflect the years lived in good and poor health. The first is HALE, whose estimation was based on Sullivan´s method, using multiple-decrement life tables and estimated YLD per capita [19–21]. On the other hand, the percentage of years of life spent in poor health was obtained, *i*.*e*., the time of total life expectancy that was not spent in good health [1]. Details on the calculation procedures can be found in previous studies [1, 11, 14, 19–21].

### 2.2 Analysis procedure

First, information on LE by sex and state in 1990, 2010, and 2019 is shown. The same information is shown for HALE, as well as being disaggregated by age group. The relative percentage change in the indicators was calculated between the periods 1990–2010 and 2010–2019, as well as the absolute percentage change (1990–2019). A Pearson correlation analysis (*r*) was made to evaluate the relationship between HALE at the state level with the Socio Demographic Index (SDI) and with the Healthcare Access and Quality Index (HAQ). Likewise, the *p-value* was estimated through the Chi-Square test. The SDI is a composite average of the rankings of per capita income, average educational level, and fertility rates in a given population and year [26]. The index ranges from 0 to 1, which means that the lower the SDI value, the lower the development index, while a value of 1 indicates a higher level of socio demographic development [26]. On the other hand, the HAQ includes preventable causes of death that should not have occurred if timely and effective care had been available [27]. This index is given on a scale from 0 to 100, where 0 represents the worst level observed, and 100 is the best [27]. The information was organized and processed with the statistical program Stata®17 [28].

### 2.3 Ethical considerations

The authors did not directly collect consent to participate because this study is a secondary data analysis research, but all research was carried out in accordance with relevant national and international guidelines and regulations. This study uses publicly available secondary information, available at: https://vizhub.healthdata.org/gbd-compare/# [29]. All data used and analyzed during the current study are available from the corresponding authors upon reasonable request (mgiraldo@inger.gob.mx; magudelo@unam.mx). The database does not contain individual identifiers. The GBD uses a standardized and replicable approach in compliance with the Guidelines for Accurate and Transparent Health Estimates Reporting (GATHER) [22, 23].

## 3. Results

### 3.1 LE and HALE by sex and state

At the national level, the LE increased from 70.9 years in 1990 to 74.3 years in 2000 and to 75.6 years in 2019, meaning an increase of 6.7% between 1990 and 2019. For men, this indicator was 68.3, 71.9, and 72.7 years in 1990, 2010, and 2019, respectively, while it was 73.5 years (1990), 76.6 years (2010), and 78.6 years (2019) for women. In turn, HALE increased by 6.4% for both sex (6.1% for men and 6.4% for women) between 1990 and 2019. In 2019, this indicator was 65.4 years for the entire Mexican population, 63.9 years for men, and 66.9 years for women. As can be observed, both indicators were higher for the female group than for the male group in the years evaluated.

Table 1 shows the LE and HALE values for men and women in each state for the three years considered. Both men and women were found to have increases in both indicators, but with notable differences. For example, LE and HALE were higher for women than men in all years and all states of the country; however, the patterns of these indicators varied. Such is the case of men and women in Colima, who had the lowest LE in the country in 2019, while men in Jalisco and women in Sonora had the highest value. Between 1990 and 2019, men in Oaxaca had the highest gain in years of life at birth, and those in Quintana Roo had the lowest value. On the other hand, women in Nuevo León had the highest gain in LE, compared to women in Colima, who only had a gain of 1.4 years of life (1.9%). Furthermore, the upward trend observed in LE between 1990 and 2010 was slowed and even reduced for men in states such as Ciudad de México (1.8 years), Guerrero (1. 8 years), Morelos (0.9 years), Quintana Roo (1.9 years), San Luis Potosí (0.2 years), Sinaloa (0.5 years), Tabasco (0.1 years), Tamaulipas (1 year), Yucatán (0.3 years) and Zacatecas (0.5 years) in the period between 2010 and 2019.

**Table 1:** Life expectancy and health-adjusted life expectancy by sex and state. Mexico, 1990, 2010 and 2019.

**Males**														
**States**	**Life expectancy**							**Health-adjusted life expectancy**						
	**1990**	**2010**	**2019**	**% Change** **1990–2009**	**% Change** **2010–2019**	**% Change** **1990–2019**		**1990**	**2010**	**2019**	**% Change** **1990–2009**	**% Change** **2010–2019**	**% Change** **1990–2019**	
Aguascalientes	70.0	72.9	74.5	4.1	2.3	6.5		61.7	65.3	65.5	5.9	0.3	6.2	
Baja California	66.2	67.6	69.9	2.1	3.4	5.5		58.6	61.7	61.7	5.4	0.0	5.4	
Baja California Sur	69.6	71.9	73.6	3.2	2.5	5.7		61.6	64.8	64.8	5.2	-0.1	5.2	
Campeche	69.3	73.5	74.0	6.1	0.6	6.8		60.9	65.1	64.9	6.9	-0.3	6.5	
Coahuila	68.0	71.2	73.8	4.7	3.7	8.6		60.1	63.9	64.7	6.2	1.4	7.7	
Colima	64.9	68.2	68.5	5.0	0.5	5.5		59.2	64.4	61.7	8.8	-4.3	4.1	
Chiapas	69.6	72.1	72.8	3.7	0.9	4.6		59.9	64.6	64.9	7.8	0.6	8.5	
Chihuahua	68.0	71.4	73.5	5.1	2.9	8.1		57.3	55.4	60.4	-3.2	8.9	5.4	
Ciudad de México	66.9	71.8	70.0	7.3	-2.5	4.6		61.1	64.0	63.7	4.8	-0.5	4.3	
Durango	68.4	73.1	75.0	7.0	2.5	9.6		60.2	62.0	65.9	3.0	6.3	9.4	
Guanajuato	67.5	71.4	72.9	5.8	2.1	8.1		60.3	65.1	62.4	7.9	-4.1	3.5	
Guerrero	68.4	72.7	70.9	6.4	-2.5	3.7		58.4	62.5	63.7	6.9	1.9	9.0	
Hidalgo	65.9	70.1	72.4	6.4	3.2	9.9		60.8	65.7	66.1	8.1	0.6	8.7	
Jalisco	69.0	74.1	75.2	7.4	1.5	8.9		60.4	64.2	63.4	6.3	-1.2	5.0	
Estado de México	68.5	71.6	72.1	4.5	0.7	5.2		59.4	64.3	63.9	8.3	-0.7	7.5	
Michoacán	69.4	72.6	73.3	4.7	0.9	5.7		61.0	64.8	64.4	6.2	-0.6	5.5	
Morelos	68.3	72.8	71.9	6.6	-1.2	5.3		60.3	65.0	63.4	7.7	-2.4	5.2	
Nayarit	68.9	73.8	74.7	7.2	1.2	8.4		60.6	63.9	65.5	5.5	2.5	8.1	
Nuevo León	70.4	73.0	73.8	3.7	1.1	4.8		62.4	64.9	65.3	4.0	0.5	4.6	
Oaxaca	66.4	71.5	73.8	7.6	3.3	11.2		58.5	64.4	64.9	10.2	0.7	11.0	
Puebla	65.8	69.9	71.9	6.3	2.9	9.3		58.1	64.1	63.4	10.2	-1.1	9.0	
Querétaro	67.2	71.1	73.3	5.9	3.0	9.1		59.4	64.9	64.7	9.3	-0.4	8.8	
Quintana Roo	70.7	73.6	71.6	4.0	-2.6	1.3		62.2	64.3	63.0	3.4	-2.1	1.3	
San Luis Potosí	70.4	74.1	73.9	5.3	-0.3	5.0		61.9	65.8	65.0	6.2	-1.1	5.0	
Sinaloa	70.7	73.6	73.1	4.0	-0.7	3.3		62.4	62.4	64.4	0.0	3.1	3.2	
Sonora	68.2	70.6	71.3	3.4	1.1	4.6		60.4	63.0	63.1	4.4	0.0	4.4	
Tabasco	68.6	72.2	72.1	5.3	-0.1	5.2		60.3	63.1	63.3	4.8	0.2	5.0	
Tamaulipas	69.4	73.4	72.4	5.8	-1.3	4.3		61.2	63.5	63.9	3.8	0.5	4.3	
Tlaxcala	68.8	72.7	73.5	5.6	1.1	6.7		60.7	65.5	64.7	7.9	-1.4	6.5	
Veracruz	69.0	72.5	73.0	5.2	0.6	5.9		60.7	64.2	64.1	5.9	-0.3	5.6	
Yucatán	70.6	73.8	73.5	4.5	-0.4	4.1		62.6	65.5	64.9	4.7	-0.9	3.8	
Zacatecas	71.3	73.8	73.3	3.5	-0.7	2.8		62.7	65.0	64.4	3.7	-1.0	2.7	
**Females**														
**States**	**Life expectancy**							**Health-adjusted life expectancy**						
	**1990**	**2010**	**2019**	**% Change** **1990–2009**	**% Change** **2010–2019**	**% Change** **1990–2019**		**1990**	**2010**	**2019**	**% Change** **1990–2009**	**% Change** **2010–2019**	**% Change** **1990–2019**	
Aguascalientes	73.0	76.6	79.2	4.9	3.3	8.4		63.2	66.4	67.3	5.0	1.3	6.4	
Baja California	75.7	75.0	78.2	-0.9	4.2	3.3		62.5	65.9	66.7	5.5	1.2	6.8	
Baja California Sur	73.4	77.6	78.8	5.6	1.6	7.3		64.3	66.6	67.0	3.5	0.7	4.2	
Campeche	71.2	76.9	78.3	8.1	1.8	10.0		62.5	66.2	66.4	5.9	0.4	6.3	
Coahuila	71.3	74.8	76.8	4.8	2.7	7.7		61.9	65.7	66.1	6.1	0.6	6.7	
Colima	74.7	74.2	76.2	-0.7	2.6	1.9		61.9	66.7	67.1	7.8	0.5	8.4	
Chiapas	72.6	76.8	78.8	5.8	2.5	8.4		60.8	65.6	65.5	7.7	-0.2	7.6	
Chihuahua	72.6	75.5	77.6	3.9	2.8	6.8		60.7	63.6	64.7	4.8	1.7	6.5	
Ciudad de México	74.1	76.8	78.9	3.6	2.8	6.6		63.8	66.4	67.0	4.2	0.8	5.0	
Durango	73.2	77.7	80.0	6.2	2.9	9.3		63.1	66.6	67.9	5.4	2.0	7.5	
Guanajuato	73.4	75.8	78.4	3.2	3.4	6.7		62.7	66.9	66.8	6.7	-0.1	6.6	
Guerrero	72.8	77.1	78.5	6.0	1.7	7.9		62.3	67.1	68.8	7.7	2.6	10.5	
Hidalgo	74.5	75.9	81.2	2.0	6.9	9.0		63.4	68.1	68.1	7.4	0.0	7.4	
Jalisco	73.3	78.7	80.0	7.3	1.7	9.1		62.6	66.5	66.8	6.2	0.6	6.8	
Estado de México	75.1	76.1	78.6	1.2	3.4	4.6		62.2	65.9	66.4	6.0	0.7	6.7	
Michoacán	74.0	77.6	80.2	5.0	3.3	8.4		64.1	67.6	68.3	5.5	1.1	6.6	
Morelos	74.6	77.2	78.6	3.5	1.7	5.3		63.2	66.9	67.0	5.9	0.1	6.0	
Nayarit	74.7	78.1	80.7	4.6	3.3	8.0		63.6	67.7	68.6	6.5	1.2	7.8	
Nuevo León	71.6	77.0	79.3	7.6	2.9	10.7		64.0	67.4	67.7	5.3	0.4	5.8	
Oaxaca	71.2	76.5	79.1	7.4	3.5	11.2		61.2	67.3	67.5	10.0	0.4	10.4	
Puebla	72.9	75.4	77.7	3.4	3.1	6.5		60.9	66.0	66.3	8.4	0.4	8.9	
Querétaro	76.0	76.6	78.9	0.8	3.1	3.8		62.4	67.0	67.4	7.4	0.6	8.0	
Quintana Roo	74.8	77.2	77.3	3.3	0.1	3.4		64.6	65.9	65.6	2.0	-0.5	1.5	
San Luis Potosí	76.2	78.1	79.5	2.5	1.7	4.3		63.9	67.8	67.8	6.2	0.0	6.2	
Sinaloa	73.7	78.9	81.3	7.1	3.0	10.3		65.1	68.3	69.1	5.0	1.2	6.2	
Sonora	72.9	75.9	78.2	4.1	3.1	7.3		62.7	65.5	66.4	4.6	1.4	6.0	
Tabasco	74.4	76.3	77.3	2.5	1.3	3.8		62.1	65.4	65.6	5.4	0.3	5.7	
Tamaulipas	72.8	77.5	79.3	6.5	2.3	8.9		63.3	66.9	67.2	5.7	0.4	6.1	
Tlaxcala	74.0	76.7	78.9	3.6	3.0	6.6		62.1	66.5	67.1	7.1	0.9	8.1	
Veracruz	73.9	77.4	78.1	4.8	0.9	5.7		62.8	66.0	66.3	5.1	0.4	5.5	
Yucatán	75.4	76.7	77.8	1.6	1.5	3.1		63.3	66.5	66.4	5.1	-0.1	5.0	
Zacatecas	75.4	77.9	79.5	3.3	2.0	5.4		64.3	67.1	67.6	4.4	0.8	5.2	
	100	70	20											
	High	Medium	Low											

By 2019, the states with the highest and lowest HALE were Hidalgo (66.1 years) and Chihuahua (60.0 years) for men and Sinaloa (69.1 years) and Chiapas (65.5 years) for women. Men in Oaxaca had the highest increase in HALE (11.0%), and both men and women in Quintana Roo had the lowest (1.3% and 1.5%, respectively). Women in Puebla had an increase of 8.9% in their HALE, which was the highest increase reported in the period 1990–2019. For the last year of study (2019), several states were one year or more below the national average HALE: Chihuahua, 3.5 years; Baja California, 2.2 years; Colima, 2.2 years; and Guanajuato, 1.5 years for men and Chihuahua, 2.2 years; Chiapas, 1.4 years; Quintana Roo, 1.3 years; and Tabasco, 1.3 years for women. Between 1990 and 2010, in all states, there was an increase in HALE for both sex (except for men in Chihuahua). However, this gain reversed for men in 16 states (Campeche, Colima, Ciudad de México, Guanajuato, Jalisco, Estado de Mexico, Michoacán, Morelos, Puebla, Querétaro, Quintana Roo, San Luis Potosí, Tlaxcala, Veracruz, Yucatán, and Zacatecas) for the period 2010–2019. Among women, the same trend occurred in Chiapas, Guanajuato, Quintana Roo, and Yucatan (Table 1).

### 3.2 Differences in LE and HALE by state (both sex), and poor health by state and sex

**Fig 1:**
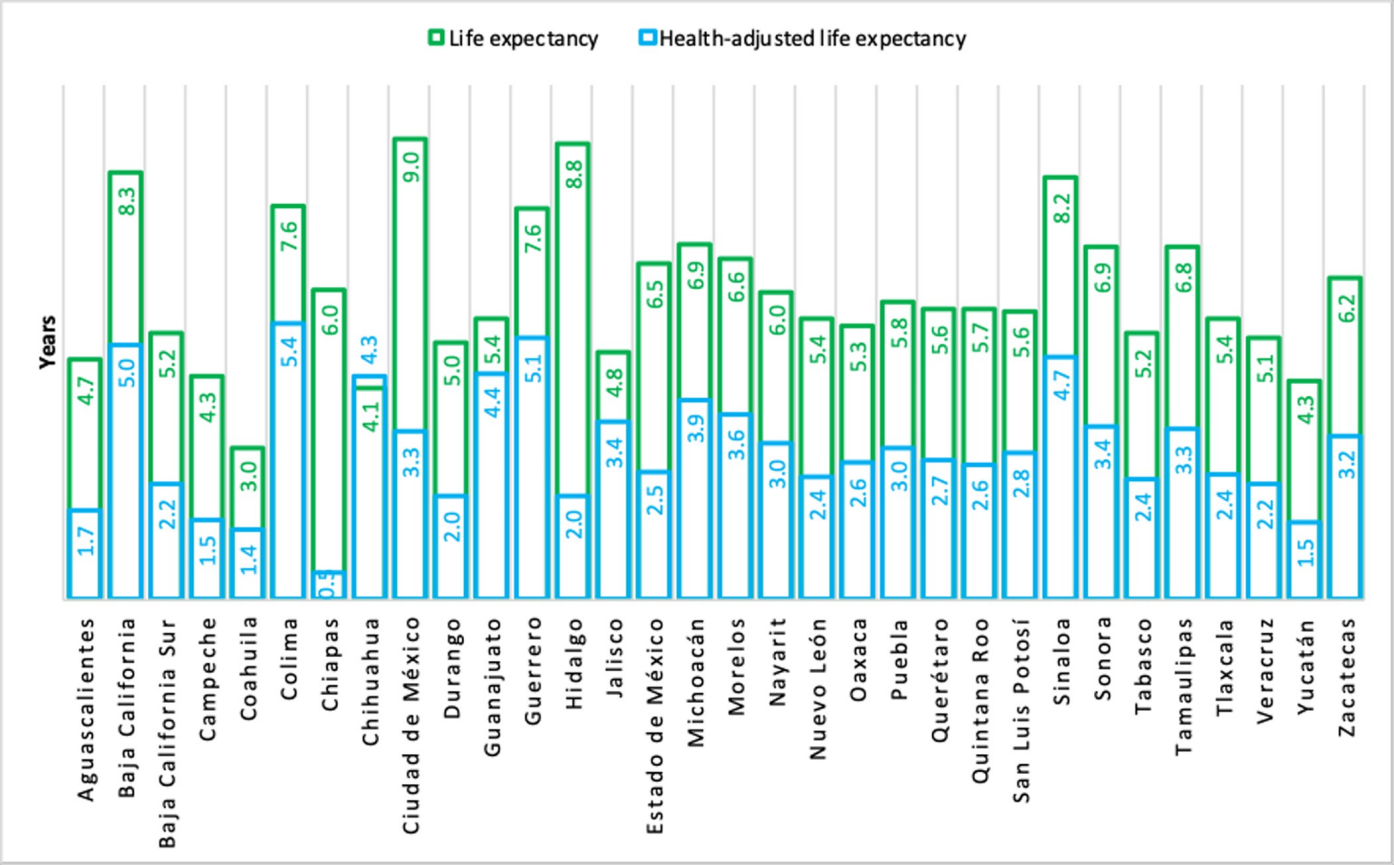
Differences in life expectancy and health-adjusted life expectancy by state (both sex). México, 2019.

The differences in the LE and HALE indicators by state for the entire population in 2019 is shown in Fig 1. States such as Hidalgo, Ciudad de México, Chiapas, and Estado de México, had a difference between LE and HALE of 4 years or more. Meanwhile, in states such as Chihuahua, Guanajuato, Jalisco, Coahuila and Colima, there was a narrower gap (less than two years) between these indicators.

**Fig 2:**
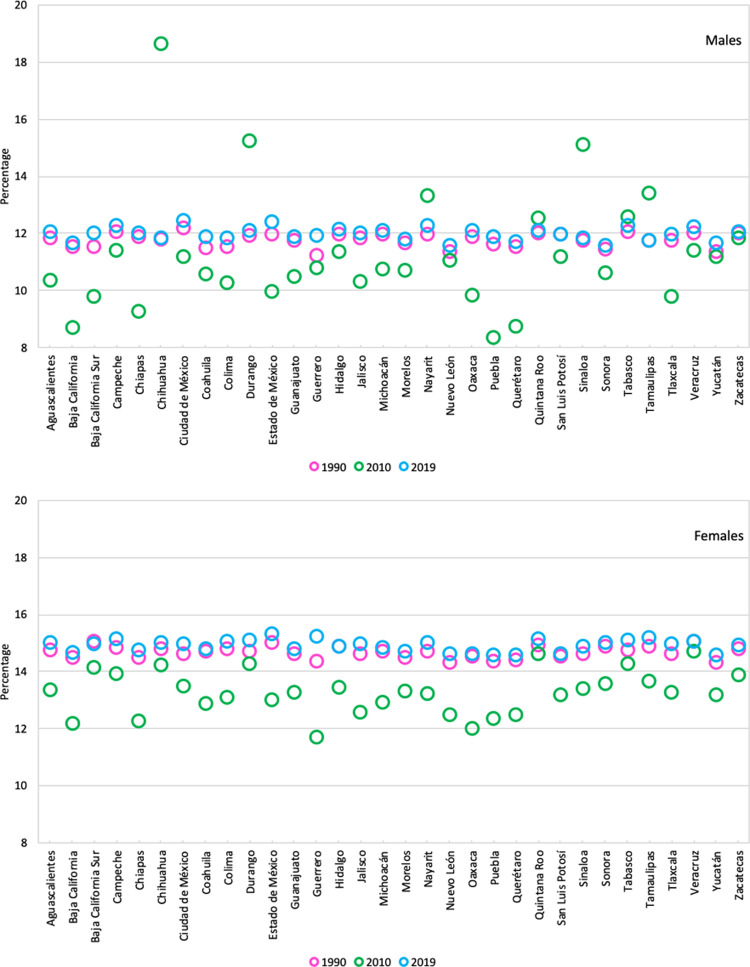
Percentage of years lived in poor health by sex and state. México, 1990, 2010 and 2019.

In percentage terms, women were found to spend more of their LE in poor health than men, as shown in Fig 2. In 2019, Mexican women spent 15% of their total LE, which translates to 11.8 years of life, in poor health or with some disability. For men, the percentage was lower as 12.1% of their total LE, which means 8.8 years, were in poor health.

Also, in 2019, this percentage ranged between 11.7% and 12.5% for men and between 14.6% and 15.3% for women by state. It should be noted that the percentage of years lived in poor health decreased in most states (except for men in Durango, Nayarit, Quintana Roo, Sinaloa, Tabasco, and Tamaulipas) in 2010. However, by 2019, virtually all states returned to the values reported in 1990.

### 3.3 HALE by sex and age group

Table 2 shows the HALE indicator by sex and age group. It stands out that the most significant gains in healthy life years occurred in the first period (1990–2010); also, in the following period (2010–2019), the male group experienced a slight reduction in almost all age groups, while the female group had an emerging increase. Between 1990 and 2019, the most relevant increases in this indicator occurred in the most advanced age groups (55 years and older). Children under 1 year of age gained the most years of healthy life during the study period: 3.7 years for males and 4.2 years for females.

**Table 2:** Health-adjusted life expectancy by sex and age group. Mexico, 1990, 2010 and 2019.

Age groups	Males					
	1990	2010	2019	% Change 1990–2010	% Change 2010–2019	% Change 1990–2019
<1	60.2	63.9	63.9	6.2	0.0	6.1
1–4	61.8	64.2	63.8	3.9	-0.5	3.3
5–9	58.5	60.5	60.1	3.5	-0.7	2.8
10–14	53.8	55.8	55.3	3.6	-0.8	2.8
15–19	49.2	51.1	50.6	3.8	-0.9	2.9
20–24	44.8	46.7	46.2	4.1	-1.0	3.1
25–29	40.6	42.4	42.0	4.5	-1.0	3.4
30–34	36.5	38.3	37.9	4.9	-1.0	3.8
35–39	32.5	34.2	33.9	5.3	-1.0	4.2
40–44	28.6	30.2	29.9	5.8	-1.1	4.7
45–49	24.7	26.3	26.1	6.3	-0.9	5.3
50–54	21.1	22.6	22.4	6.8	-0.9	5.9
55–59	17.7	19.1	18.9	7.7	-0.8	6.8
60–64	14.6	15.8	15.7	8.6	-0.6	7.9
65–69	11.7	12.9	12.8	9.9	-0.3	9.6
70–74	9.2	10.2	10.2	11.1	0.2	11.3
75–79	6.9	7.9	7.9	14.5	-0.1	14.3
**Age groups**	**Females**					
	**1990**	**2010**	**2019**	**% Change 1990–2010**	**% Change 2010–2019**	**% Change 1990–2019**
<1	62.7	66.4	66.9	6.0	0.6	6.7
1–4	63.8	66.5	66.6	4.3	0.2	4.5
5–9	60.5	62.8	62.9	3.9	0.1	4.1
10–14	55.8	58.1	58.2	4.1	0.1	4.2
15–19	51.2	53.4	53.5	4.4	0.1	4.5
20–24	46.7	48.9	49.0	4.8	0.1	4.9
25–29	42.3	44.5	44.5	5.3	0.1	5.4
30–34	38.0	40.2	40.2	5.8	0.2	6.0
35–39	33.8	35.9	36.0	6.3	0.3	6.6
40–44	29.7	31.7	31.9	6.9	0.4	7.3
45–49	25.8	27.7	27.9	7.6	0.5	8.2
50–54	22.0	23.9	24.0	8.6	0.7	9.3
55–59	18.4	20.2	20.4	9.8	0.9	10.8
60–64	15.1	16.8	17.0	11.4	1.1	12.7
65–69	12.0	13.7	13.9	13.6	1.5	15.3
70–74	9.3	10.8	11.0	16.2	2.2	18.7
75–79	6.8	8.2	8.4	21.4	2.0	23.8

### 3.4 HALE by sex and state

By states, a varied trend in HALE by age group and sex was identified. Men in Chihuahua, Chiapas, Quintana Roo and Tabasco had the lowest values, as well as women in Baja California, Chihuahua, and Ciudad de México. On the other hand, men from Guerrero, Michoacán, Nayarit, and Sinaloa had the highest HALE, as well as women from Aguascalientes, Durango, and Hidalgo. In the oldest and youngest age groups by sex, the following differences were evident: 4.4 years of difference between the highest and lowest HALE in children under one year of age and 3.2 years in men aged 75–79 years; in women, such differences were 5.7 years for children under one year of age and 1.9 years for women aged 75–79 years (Table 3).

**Table 3:** Health-adjusted life expectancy by sex, age group and state. Mexico, 2019 (Heatmaps).

	Age groups																
States	0	1–4	5–9	10–14	15–19	20–24	25–29	30–34	35–39	40–44	45–49	50–54	55–59	60–64	65–69	70–74	75–79
	**Males**																
Aguascalientes	67.3	67.0	63.3	58.5	53.8	49.2	44.8	40.5	36.3	32.2	28.1	24.2	20.5	17.0	13.8	10.9	8.3
Baja California	66.7	66.3	62.6	57.8	53.1	48.6	44.2	39.9	35.7	31.6	27.7	23.8	20.2	16.7	13.5	10.6	8.0
Baja California Sur	67.0	66.8	63.0	58.3	53.6	49.1	44.6	40.3	36.1	31.9	27.9	24.0	20.4	16.9	13.7	10.8	8.0
Campeche	66.4	66.3	62.6	57.8	53.1	48.6	44.2	39.9	35.7	31.5	27.5	23.7	20.1	16.7	13.7	10.8	8.2
Coahuila	66.1	66.0	62.2	57.5	52.8	48.2	43.8	39.4	35.2	31.0	27.1	23.2	19.6	16.3	13.2	10.5	8.0
Colima	67.1	66.6	62.9	58.2	53.5	49.0	44.6	40.3	36.1	32.0	28.1	24.2	20.5	17.1	14.0	11.0	8.3
Chiapas	65.5	65.2	61.6	56.9	52.3	47.7	43.4	39.0	34.8	30.7	26.8	23.0	19.6	16.3	13.3	10.5	7.9
Chihuahua	64.7	64.7	61.0	56.3	51.6	47.2	42.8	38.6	34.4	30.3	26.4	22.6	19.0	15.7	12.7	10.0	7.6
Ciudad de México	67.0	66.8	63.0	58.2	53.5	49.0	44.6	40.3	36.0	31.9	27.9	24.1	20.4	16.9	13.8	10.9	8.2
Durango	67.9	67.7	64.0	59.2	54.5	50.0	45.5	41.2	36.9	32.8	28.8	24.9	21.3	17.9	14.8	11.9	9.5
Guanajuato	66.8	66.6	62.8	58.1	53.4	48.9	44.5	40.2	35.9	31.8	27.8	23.9	20.2	16.8	13.7	10.8	8.1
Guerrero	68.8	68.6	65.0	60.3	55.6	51.1	46.7	42.5	38.2	34.1	30.1	26.3	22.7	19.4	16.2	13.3	10.6
Hidalgo	68.1	67.7	64.0	59.2	54.6	50.0	45.6	41.3	37.0	32.9	28.9	25.0	21.3	17.9	14.6	11.7	9.0
Jalisco	66.8	66.6	62.8	58.1	53.4	48.9	44.5	40.2	36.0	31.8	27.8	24.0	20.3	16.8	13.7	10.8	8.2
México	66.4	66.1	62.4	57.6	52.9	48.4	44.0	39.6	35.4	31.3	27.3	23.5	19.9	16.5	13.4	10.6	8.0
Michoacán	68.3	68.0	64.2	59.5	54.8	50.3	45.9	41.6	37.3	33.2	29.1	25.2	21.5	18.0	14.8	11.9	9.2
Morelos	67.0	66.7	63.0	58.2	53.5	49.0	44.6	40.2	36.0	31.9	27.8	24.0	20.4	16.9	13.8	11.0	8.4
Nayarit	68.6	68.2	64.6	59.8	55.1	50.5	46.2	41.9	37.7	33.5	29.5	25.5	21.8	18.4	15.1	12.1	9.4
Nuevo León	67.7	67.3	63.7	58.9	54.3	49.7	45.2	40.9	36.6	32.4	28.4	24.4	20.7	17.2	14.0	11.1	8.4
Oaxaca	67.5	67.2	63.5	58.8	54.1	49.6	45.1	40.8	36.6	32.5	28.5	24.7	21.0	17.6	14.5	11.6	8.9
Puebla	66.3	66.2	62.6	57.8	53.2	48.6	44.2	39.9	35.6	31.5	27.5	23.6	20.0	16.6	13.6	10.8	8.1
Querétaro	67.4	67.1	63.4	58.6	53.9	49.4	45.0	40.6	36.4	32.2	28.1	24.2	20.5	17.1	13.9	11.0	8.2
Quintana Roo	65.6	65.2	61.6	56.8	52.1	47.6	43.2	38.9	34.6	30.5	26.5	22.7	19.1	15.7	12.7	9.9	7.4
San Luis Potosí	67.8	67.4	63.8	59.0	54.3	49.8	45.4	41.0	36.8	32.6	28.6	24.7	21.0	17.6	14.4	11.4	8.7
Sinaloa	69.1	68.8	65.1	60.4	55.7	51.1	46.7	42.3	38.1	33.9	29.9	25.9	22.2	18.6	15.3	12.3	9.5
Sonora	66.4	66.2	62.5	57.7	53.1	48.5	44.1	39.8	35.6	31.4	27.5	23.6	19.9	16.5	13.3	10.5	7.9
Tabasco	65.6	65.5	61.9	57.1	52.5	47.9	43.5	39.2	35.0	30.9	27.0	23.2	19.6	16.3	13.3	10.6	8.0
Tamaulipas	67.2	67.0	63.2	58.5	53.8	49.3	44.9	40.5	36.3	32.2	28.2	24.4	20.8	17.4	14.4	11.6	9.0
Tlaxcala	67.1	67.0	63.3	58.5	53.8	49.3	44.9	40.6	36.3	32.2	28.1	24.3	20.6	17.2	14.1	11.2	8.6
Veracruz	66.3	66.0	62.4	57.6	53.0	48.4	44.0	39.7	35.5	31.4	27.4	23.6	20.0	16.7	13.6	10.9	8.3
Yucatán	66.4	66.2	62.5	57.7	53.0	48.5	44.0	39.7	35.5	31.3	27.3	23.4	19.8	16.4	13.3	10.5	7.8
Zacatecas	67.6	67.3	63.6	58.9	54.2	49.7	45.3	41.0	36.7	32.6	28.6	24.7	21.0	17.6	14.4	11.5	8.7
	**Females**																
Aguascalientes	65.5	65.4	61.7	56.9	52.2	47.8	43.5	39.2	35.0	30.9	27.0	23.1	19.5	16.1	13.1	10.3	7.9
Baja California	61.7	61.5	57.7	53.0	48.2	43.8	39.8	35.8	32.0	28.2	24.6	21.1	17.9	14.9	12.2	9.7	7.5
Baja California Sur	64.8	64.6	60.9	56.2	51.4	46.9	42.6	38.4	34.3	30.2	26.2	22.3	18.7	15.4	12.4	9.7	7.5
Campeche	64.9	65.0	61.3	56.5	51.8	47.4	43.1	38.9	34.7	30.7	26.8	23.0	19.4	16.2	13.3	10.5	8.2
Coahuila	64.7	64.8	61.0	56.3	51.6	47.0	42.6	38.4	34.1	30.0	26.0	22.2	18.6	15.4	12.5	10.0	7.8
Colima	61.7	61.3	57.6	52.9	48.2	43.8	39.9	36.3	32.6	29.1	25.5	22.0	18.6	15.4	12.6	10.0	7.7
Chiapas	64.9	64.9	61.2	56.5	51.8	47.3	43.0	38.8	34.7	30.7	26.8	23.1	19.6	16.4	13.3	10.5	8.0
Chihuahua	60.4	60.5	56.9	52.1	47.4	43.2	39.2	35.3	31.4	27.7	24.0	20.5	17.2	14.1	11.4	9.0	7.0
Ciudad de México	63.7	63.6	59.9	55.1	50.4	45.9	41.6	37.5	33.4	29.4	25.5	21.9	18.5	15.3	12.5	10.0	7.6
Durango	65.9	65.8	62.1	57.3	52.7	48.2	43.8	39.6	35.4	31.2	27.3	23.5	20.0	16.6	13.7	11.1	8.9
Guanajuato	62.4	62.3	58.6	53.8	49.1	44.8	40.9	37.1	33.2	29.4	25.7	22.1	18.7	15.5	12.6	10.0	7.7
Guerrero	63.7	63.6	60.1	55.3	50.6	46.3	42.3	38.4	34.5	30.8	27.1	23.5	20.0	16.8	13.7	10.9	8.3
Hidalgo	66.1	65.8	62.1	57.4	52.7	48.2	43.8	39.6	35.5	31.4	27.4	23.6	20.1	16.8	13.9	11.2	8.7
Jalisco	63.4	63.3	59.6	54.8	50.2	45.7	41.6	37.6	33.6	29.6	25.8	22.1	18.6	15.4	12.5	9.9	7.7
México	63.9	63.8	60.0	55.3	50.6	46.1	41.8	37.7	33.6	29.6	25.7	22.0	18.6	15.4	12.5	10.0	7.7
Michoacán	64.4	64.2	60.5	55.7	51.0	46.7	42.7	38.7	34.8	31.0	27.3	23.7	20.2	17.0	14.0	11.3	8.8
Morelos	63.4	63.3	59.5	54.7	50.0	45.6	41.6	37.7	33.7	29.8	26.1	22.4	19.0	15.9	12.9	10.4	8.0
Nayarit	65.5	65.3	61.6	56.9	52.2	47.8	43.6	39.5	35.5	31.5	27.7	23.8	20.2	16.9	13.9	11.1	8.9
Nuevo León	65.3	65.2	61.5	56.7	52.0	47.5	43.1	38.8	34.5	30.4	26.3	22.5	18.9	15.6	12.7	10.1	7.8
Oaxaca	64.9	64.6	61.0	56.2	51.5	47.0	42.8	38.7	34.7	30.8	27.0	23.4	20.0	16.8	13.8	11.1	8.6
Puebla	63.4	63.4	59.8	55.0	50.4	45.9	41.6	37.5	33.5	29.5	25.7	22.0	18.6	15.6	12.7	10.1	7.8
Querétaro	64.7	64.6	60.9	56.1	51.4	46.9	42.6	38.4	34.2	30.2	26.3	22.5	18.9	15.7	12.8	10.1	7.7
Quintana Roo	63.0	62.7	59.1	54.4	49.7	45.3	41.3	37.2	33.3	29.3	25.5	21.8	18.2	15.0	12.1	9.6	7.3
San Luis Potosí	65.0	64.8	61.1	56.3	51.6	47.2	43.0	38.9	34.8	30.8	26.9	23.1	19.6	16.4	13.4	10.7	8.3
Sinaloa	64.4	64.3	60.5	55.8	51.1	46.7	42.5	38.5	34.5	30.5	26.5	22.7	19.1	15.7	12.7	10.1	7.9
Sonora	63.1	62.9	59.2	54.5	49.8	45.3	41.0	37.0	32.9	29.1	25.3	21.6	18.1	14.9	12.1	9.5	7.3
Tabasco	63.3	63.3	59.8	55.1	50.4	45.9	41.8	37.8	33.8	29.9	26.1	22.3	18.9	15.8	12.9	10.3	8.0
Tamaulipas	63.9	63.6	60.1	55.3	50.6	46.2	41.9	37.7	33.5	29.4	25.5	21.7	18.2	15.1	12.3	9.8	7.8
Tlaxcala	64.7	64.8	61.1	56.3	51.6	47.1	42.8	38.7	34.5	30.4	26.5	22.8	19.3	16.0	13.1	10.5	8.2
Veracruz	64.1	63.9	60.2	55.5	50.8	46.3	42.1	37.9	33.9	30.0	26.1	22.4	19.0	15.9	13.0	10.5	8.1
Yucatán	64.9	64.9	61.1	56.4	51.7	47.1	42.7	38.4	34.2	30.2	26.3	22.5	19.0	15.8	12.8	10.1	7.8
Zacatecas	64.4	64.4	60.6	55.9	51.2	46.9	42.9	39.0	35.0	31.1	27.3	23.5	19.9	16.6	13.5	10.7	8.3
																	
	High rank				Low rank												

### 3.5 Correlation between HALE with SDI and HAQ

**Fig 3:**
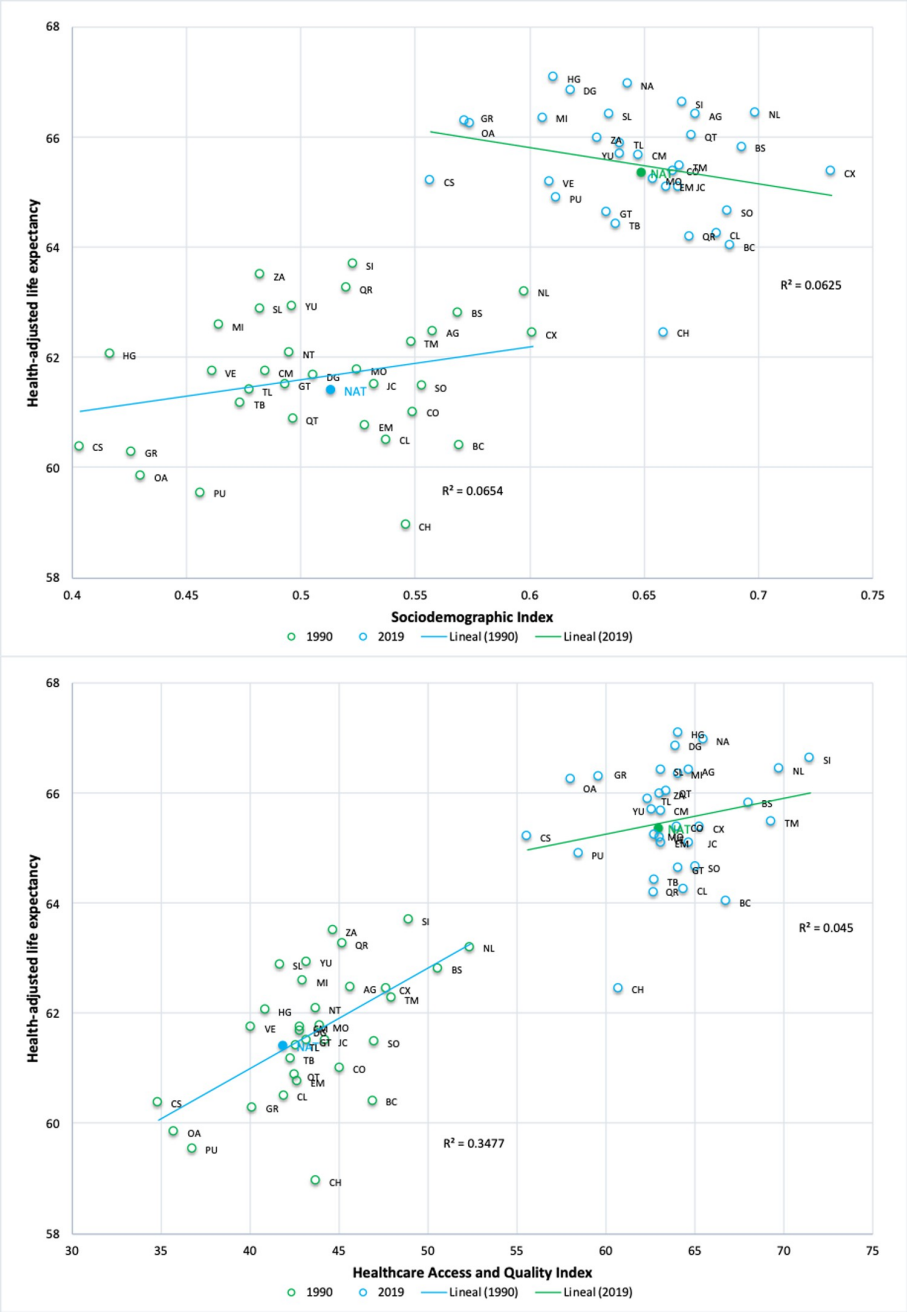
Correlation between health-adjusted life expectancy with the Socio Demographic Index and with the Healthcare Access and Quality Index, 1990 and 2019.

Fig 3 shows that SDI and HAQ increased in all states of the country between 1990 and 2019. It is worth noting that both indicators increased throughout Mexico during this period of time. In 1990, there was a positive but statistically non-significant correlation between HALE and SDI (r1990 = 0.2373; *p-value* = 0.1909), while in 2019, the correlation between both indicators was negative and also statistically non-significant (r2019 = -0.2571; *p-value* = 0.1555). In turn, a statistically significant, relatively high, and positive correlation was estimated between HALE and HAQ (r1990 = 0.5896; *p-value* = 0.0004) in 1990. However, such a relationship disappeared in 2019, which can be evidenced by the lower correlation and lack of statistical significance between both indicators (r2019 = 0.2056; *p-value* = 0.2590).

The correlation between HALE and SDI was not statistically significant for both men (r1990 = 0.1889; *p-value* = 0.2923; r2019 = -0.2646; *p-value* = 0.1367), and women (r1990 = 0.3037; *p-value* = 0.0858; r2019 = -0.1134; *p-value* = 0.5298) in both years. The correlation between HALE and HAQ was positive and statistically significant for both sex in 1990 (r1990 = 0.5165; *p-value* = 0.0021 for men; r1990 = 0.6550; *p-value* = 0.0000 for women), as well as for women in 2019 (r2019 = 0.3973; *p-value* = 0.0221) (S1 and S2 Figs).

## 4. Discussion

In this study, a pooled analysis of LE and HALE indicators based on the GBD was performed. The importance of this study lies in its scope, as the data were disaggregated by sex, age group, and state over a relatively extended period (1990–2019). Furthermore, using this type of indicators allows an alternative approach to understanding health inequalities between and within countries [8].

Some of the most relevant findings in this study were: both LE and HALE increased between 1990 and 2019, mainly among women, although they spent more years in poor health than men. By age group, the greatest gains in years of healthy life were observed in those younger than 1 year and those aged 55 years and older. At the state level, the pattern was heterogeneous, which is evidence of the diversity of life circumstances that directly influence health. At the beginning of the period (1990–2010), it is relevant that there were substantial gains in both indicators in almost all states; however, for the following period (2010–2019) these gains were lower, and in some states, there were even notable decreases.

Mexico is a country with deep social and economic contrasts that result in divergences in LE and HALE, so there is no uniform trend in the pattern of these indicators. In 2019, there was a decrease in LE for men in 10 states compared to 2010. Of particular note are Quintana Roo, where there was a decrease of 1.9 years in LE, as well as Ciudad de México and Guerrero, with a decrease of 1.8 years each. According to Gómez-Dantés et al. (2016) the leading causes of death contributing most to the loss of years of life in almost all states are violence, chronic kidney disease, and ischemic heart disease, particularly in the male group [14]. This information is consistent with the results of Aburto and Beltrán (2019), who stated that deaths of men aged 15 to 49 years had led to a slowdown in their LE; especially since 2005, when violence intensified and increased systematically [17]. In this sense, the public policies implemented to mitigate this problem have been ineffective.

Although Mexican women have a longer life span, they spend more years in worse health conditions than men. This implies that they spend 11.8 years with some disease or disability, that is, 3 years more than men. Despite this, we found that HALE decreased for men in 16 states between 2010 and 2019; Colima (2.8 years) and Guanajuato (2.6 years) were the states with the greatest setbacks. It should be noted that the same trend was observed for women in Chiapas, Guanajuato, Quintana Roo, and Yucatan, although the slowdown was almost imperceptible.

In both LE and HALE, the disparities by sex have been attributed to various factors, including different disease burden profiles gender, genetic diathesis, environment, and social norms, as well as health-related beliefs and behaviors (including the use of health services) [18–22, 30, 31]. For example, women are at greater risk of disability from different causes and of being affected by sexual and reproductive health conditions, while men are more likely to have fatal outcomes from different types of cancer, trauma, and ischemic heart disease [18–22]. It is also more socially acceptable for men to consume alcohol and tobacco, and they tend to have more aggressive and violent attitudes [31]. A gender perspective that considers the health determinants of men and women separately is necessary to achieve an impact on these indicators.

Globally, LE at birth was 73.5 years, and HALE was 63.5 years [22]. This means that Mexico exceeded the average LE by slightly more than 2 years but had a HALE almost equal to the world average [29]. In summary, these data reflect improvements of LE at birth thanks to the decrease in the overall mortality rate. However, this has not been the case with HALE, due to the sustained increase in morbidity and disability. The hypothesis underlying these results is that there has been an evident expansion of morbidity in recent years: the increase in life years has been more pronounced than the gains in HALE, resulting in more years of poor health [32–34].

As mortality has decreased, morbidity has gained relevance. Thus, in Mexico, between 1990 and 2019, the rate of years of life lost (YLL) decreased by 26.3%, while the rate of years lived with a disability (YLD) increased by 30.2% (calculated by the authors based on GBD 2019) [29]. National data reveal that chronic diseases appear from early ages and are not only manifested through a single disease, but multimorbidity (two or more diseases) is frequent in adults of different ages. Based on data from the National Health Nutrition Survey (In Spanish, Encuesta Nacional de Salud y Nutrición [ENSANUT]) 2018–2019, 12 out of every 100 Mexicans aged 20 years or older were estimated to have multimorbidity due to cardiometabolic causes, compared to 27 out of every 100 Mexicans aged 60 years or older (calculated by the authors based on ENSANUT 2018–19) [35].

On the other hand, epidemiological and demographic transitions in the states do not follow homogeneous trajectories [14, 36, 37], on the contrary, they are polarized. The main differences in morbidity and mortality are due to infectious and acute conditions, which are more prevalent in impoverished areas of the country. Evidence of this can also be seen in the gaps between chronic conditions that are common in Mexicans, such as T2D and high blood pressure [14, 37, 38]. For the period 2018–2019, the self-reported prevalence of these chronic diseases in people aged 20 years and older was 10.3% and 18.4%, respectively. As for T2D, it ranged from 7.4% (Quintana Roo) to 14% (Campeche), while the highest prevalence of high blood pressure was 26.1% (Campeche) and the lowest was 13% (Tlaxcala) [38]. In addition, Mexico as a whole is undergoing accelerated population aging, but with different intensities. Between 2000 and 2020, the population aged 60 years and older increased from 7.1 to 15.2 million, representing 7.3% and 12.0% of the total population [39]. In 2020, at the state level, the percentage of the older adult population was 7.1% in Quintana Roo and 16.2% in Ciudad de México [39].

In 2019, the difference in HALE (for both sex) determined between the state with the highest value (Hidalgo) and the state with the lowest value (Chiapas) was 4.6 years. These results can be compared with those reported in Korea [8], where the difference between the regions with the highest and lowest HALE was 3.2 years, while in Norway the difference was much smaller (1.7 years for men and 1.4 years for women) [11]. This could be explained by the fact that socioeconomic conditions are more equitable among the population in Norway, compared to Mexico, a country with major inequities. In 2020 alone, 43% of all Mexicans lived in poverty, ranging from 22.5% in Baja California, to more than 60% in states such as Oaxaca, Puebla, Guerrero, and Chiapas [40].

Another factor that may influence the territorial differences observed is the fact that the health system is segmented, fragmented, and operates under a disease-centered model of care, with few health prevention and promotion actions [41, 42]. In addition, not all people have adequate access to these health services and, in many cases, have difficulty receiving timely and quality care and treatment. For example, 28.2% of Mexicans, that is, 35.7 million people, lacked access to health services in 2020, which was lowest in Nuevo León (19.7%) and highest in Chiapas (74.4%) [43].

SDI and HALE were not statistically associated, neither in 1990 nor in 2019. In contrast, HAQ and HALE only had a positive and statistically significant correlation in 1990 for both sex and only for women in 2019 (although such correlation was much lower). In 1990, communicable and childhood diseases such as diarrheal diseases, complications of premature birth and caloric-protein deficiencies were prevalent in Mexico; many of them were highly preventable and treatable through timely medical care [14, 29]. However, the situation was different in 2019, since the health profile of Mexicans was characterized by noncommunicable diseases (ischemic heart disease, T2D, and chronic obstructive pulmonary disease) and injuries (homicides and traffic accidents) [29]. Against this backdrop, the role of health services has been limited because the control of these causes does not depend exclusively on a medical approach. In addition, these pathologies imply a high cost associated with their care and rehabilitation.

Figueroa-Lara et al. (2016) found out that only four diseases (chronic kidney disease, high blood pressure, T2D, and ischemic heart disease) accounted for 88% of the financial burden due to chronic diseases in the Secretariat of Health of Mexico [44]. There is evidence that, by eliminating the main risk factors for chronic diseases, 80% of heart disease, stroke, T2D, and more than 40% of cancer cases could be prevented [45].

Despite the significant progress achieved in terms of potentially avoidable premature mortality due to treatable causes, in 2019, the mortality rate from these causes was 117.4 per 100 000 people in Mexico, a figure higher than the average reported for Latin America (89.6) [46]. For the period 2001–2010, it was estimated that an average of 3.2 years of life were lost at the national level due to preventable causes; many of them were noncommunicable diseases that are also continuously rising among the adult population [47]. Another study assessing the progress of 30 health indicators in the country confirmed severe difficulties in achieving the SDG. Thus, 23 of such indicators were unevenly distributed, affecting the most underdeveloped states [37].

Although the data presented do not cover the period related to severe acute respiratory syndrome coronavirus 2 (SARS-CoV-2), it has been documented that this disease greatly impacted the country. In terms of LE, women lost 2.5 years and men lost 3.6 years [48]. However, estimation of the effect of this disease on HALE is recommended in future research, considering the medium and long-term sequelae that the virus generates on people´s health. In countries such as Australia, a slight decrease in HALE was found for 2020–2022 due to SARS-CoV-2 [49]. The same occurred in Scotland, where men lost 1.4 years of HALE between 2015–2017 and 2018–2020 [50]. These findings suggest that the impact caused by SARS-CoV-2 may be higher in Mexico if we consider how LE decreased and that, as of June 2023, about 7.6 million cases and 334 336 deaths had been registered [51]. In addition, a recent study showed that both the mortality rate and the SARS-CoV-2 recovery rate are highly associated with HALE at birth [6].

### 4.1 Limitations

This study has some limitations related to the estimation of LE and HALE that should be considered, despite having been described extensively in previous publications [11, 18–22, 50, 51]. First, for mortality data, the GBD quantifies the proportion of causes of death classified as junk codes, and applies corrections, and uses cause-of-death distribution methods. This explains the differences observed in the estimations of LE made by the GBD [29] and official records [15], particularly in the data reported by state level. This study used the GBD LE as a reference since it was the basis for the calculation of the HALE shown herein.

Second, the estimation of HALE can also be affected by the availability of information, *e*.*g*., underreporting, sparsity, and incomplete geographic coverage of data [20, 21]. Third, the analysis was implemented at the state level and may overlook heterogeneity within states, which may be even greater than between states. The GBD models its estimates for missing or limited data, but they are of lower reliability than those collected through robust information systems, resulting in greater uncertainty and higher confidence intervals [22, 23].

On the other hand, the definition of “good health” and what it represents may vary from place to place and between population groups, so the main limitation of the HALE is its subjective nature. However, in general terms, it is considered a reasonable measurement of morbidity [52].

Additionally, a limitation of the GBD is that it has proxy indexes to measure socioeconomic and health inequities between geographical areas, for example, the SDI and the HAQ. These indicators have been useful for assessing differences in the burden of disease between regions and countries. However, their usefulness may be limited at the subnational level because they do not consider specific aspects of the studied country such as unemployment, marginalization, social security coverage, or adequate access to health services, among others [52, 53]. Therefore, the use of social and economic indicators that better reflect these characteristics of the Mexican population is recommended for a more in-depth analysis of inequities in LE and HALE [16]. We used SDI and HAQ so that we could compare our results with those obtained in other GBD-based studies.

Finally, this paper did not consider the causes that most contributed to the loss or decrease in LE and HALE. In this study, we wanted to focus on the patterns of both indicators over time. It is evident that these indicators provide a guideline for the overall health of individuals. However, future studies should complement the analyses with YLL, YLD, and DALY data.

## 5. Conclusion

Measuring health status disparities to reduce gaps at the subnational level has been proposed as a first step. This study adds to the few analyses available on the trends of LE and HALE at the national and state levels. To the best of our knowledge, only a few studies on this topic are available. Nevertheless, most of these studies come from high-income countries, where contexts differ from those of middle-income countries, such as Mexico. Progress in LE and HALE has slowed in recent years; HALE has even had setbacks in some states. Gaps between men and women, as well as between states, are persistent. Public and population policymaking should seek to lengthen LE and focus on ensuring that such years are spent in good health and with good quality of life.

## Supporting information

S1 Fig Correlation between health-adjusted life expectancy with the Socio Demographic Index by sex, 1990 and 2019. (TIF)

S2 Fig Correlation between health-adjusted life expectancy with the Healthcare Access and Quality Index by sex, 1990 and 2019. (TIF)
