# Supplementary figures and images for: Divergences and gaps in life expectancy and health-adjusted life expectancy in Mexico: Contribution analysis of the Global Burden of Disease Study 2019
Source: PLoS One. 2023 Nov 6;18(11):e0293881. doi: 10.1371/journal.pone.0293881 (PMC10627469; doi:10.1371/journal.pone.0293881)

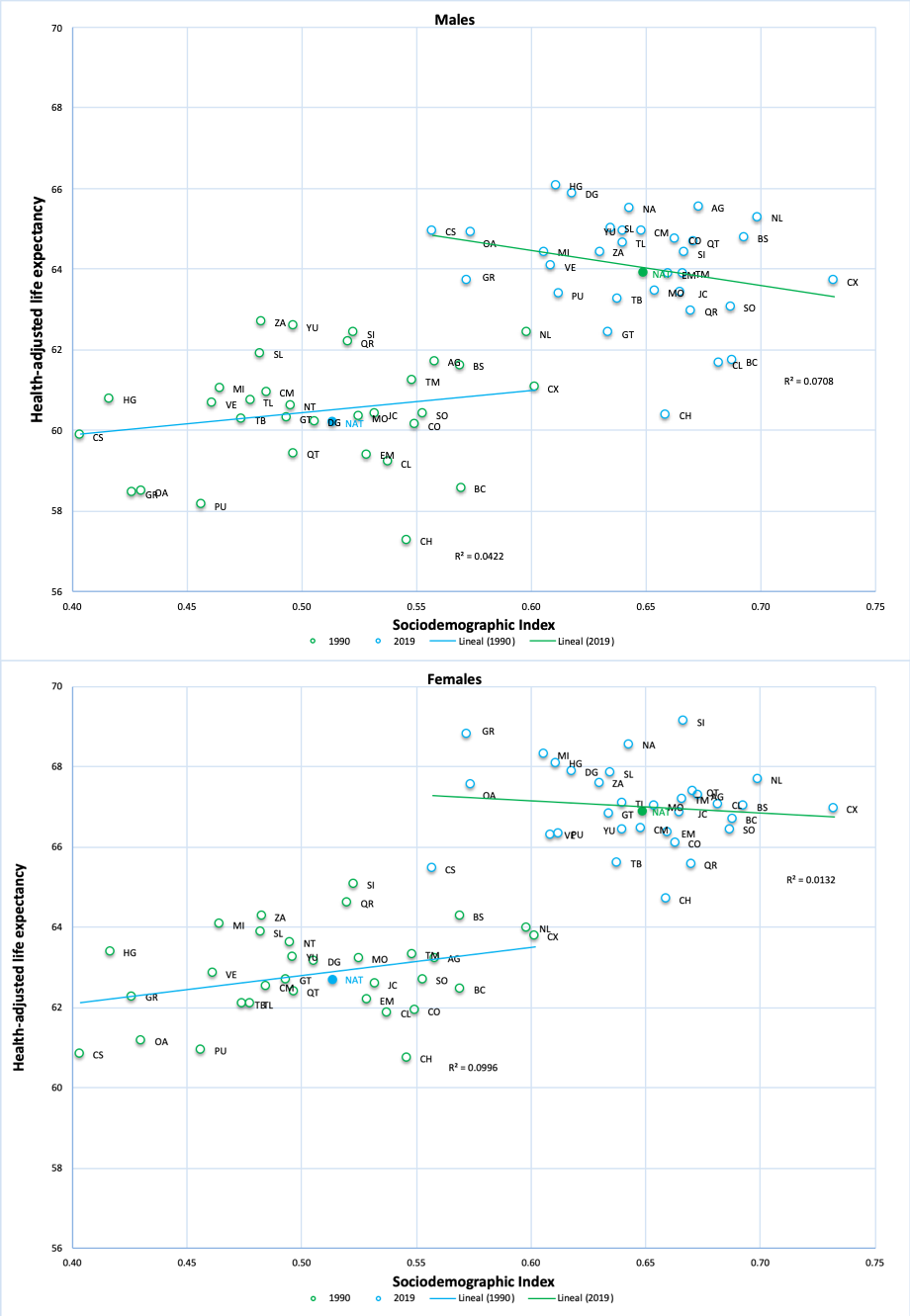

Supplement: S1 Fig — (TIF) [file pone.0293881.s001.tif]

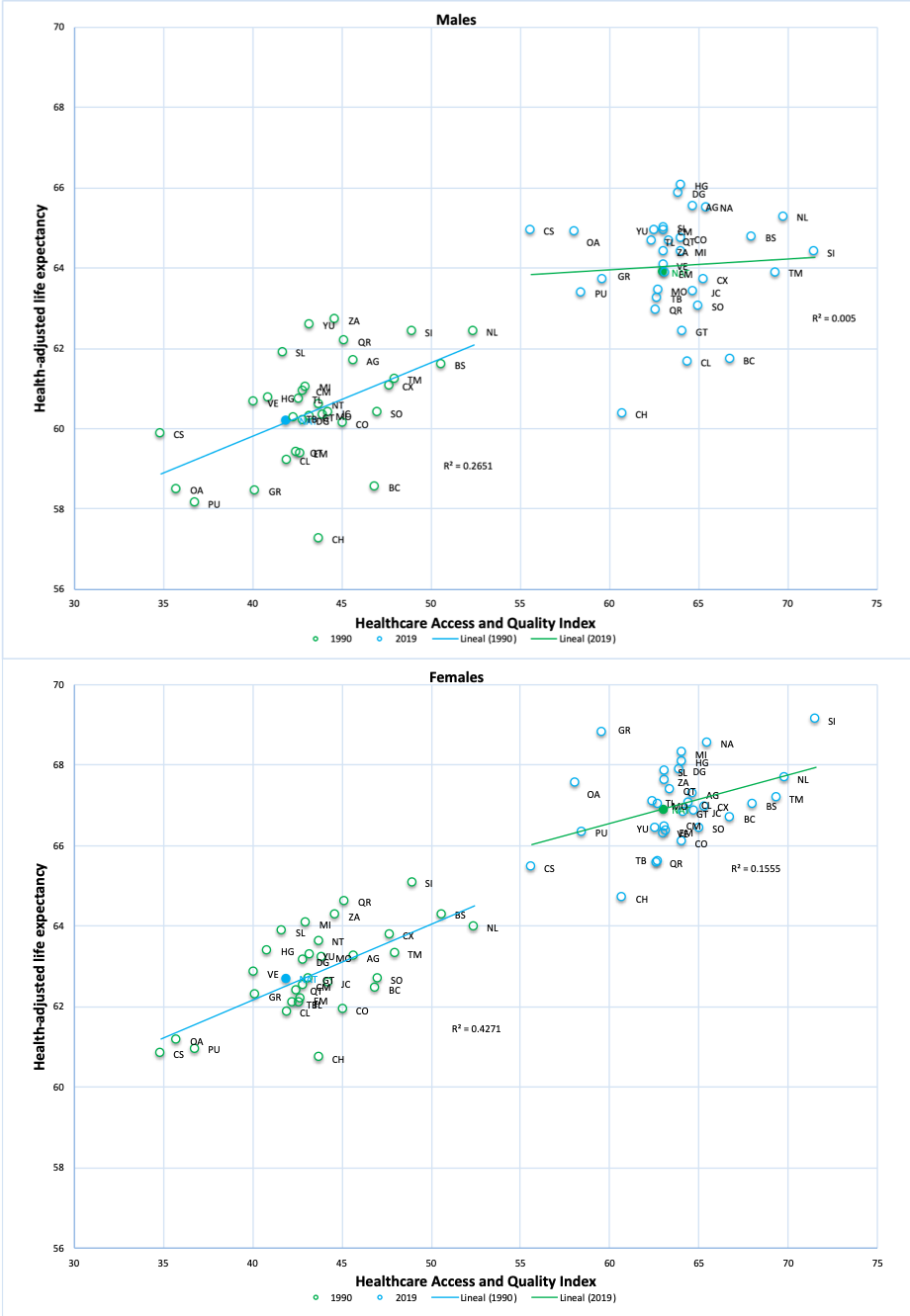

Supplement: S2 Fig — (TIF) [file pone.0293881.s002.tif]
